# Muscle glycogen level and occurrence of acid meat in commercial hybrid pigs are regulated by two low-frequency causal variants with large effects and multiple common variants with small effects

**DOI:** 10.1186/s12711-019-0488-0

**Published:** 2019-08-23

**Authors:** Xianxian Liu, Lisheng Zhou, Xianhua Xie, Zhongzi Wu, Xinwei Xiong, Zhiyan Zhang, Jie Yang, Shijun Xiao, Mengqing Zhou, Junwu Ma, Lusheng Huang

**Affiliations:** State Key Laboratory for Swine Genetics, Breeding and Production Technology, Jiangxi Agricultural University, Nanchang, 330045 China

## Abstract

**Background:**

Meat production from the commercial crossbred Duroc × (Landrace × Yorkshire) (DLY) pig is predominant in the pork industry, but its meat quality is often impaired by low ultimate pH (pHu). Muscle glycogen level at slaughter is closely associated with pHu and meat technological quality, but its genetic basis remains elusive. The aim of this study was to identify genes and/or causative mutations associated with muscle glycogen level and other meat quality traits by performing a genome-wide association study (GWAS) and additional analyses in a population of 610 DLY pigs.

**Results:**

Our initial GWAS identified a genome-wide significant (*P *= 2.54e−11) quantitative trait locus (QTL) on SSC15 (SSC for *Sus scrofa* chromosome) for the level of residual glycogen and glucose (RG) in the longissimus muscle at 45 min post-mortem. Then, we demonstrated that a low-frequency (minor allele frequency = 0.014) R200Q missense mutation in the *PRKAG3* (*RN*) gene caused this major QTL effect on RG. Moreover, we showed that the 200Q (*RN*^–^) allele was introgressed from the Hampshire breed into more than one of the parental breeds of the DLY pigs. After conditioning on R200Q, re-association analysis revealed three additional QTL for RG on SSC3 and 4, and on an unmapped scaffold (AEMK02000452.1). The SSC3 QTL was most likely caused by a splice mutation (g.8283C>A) in the *PHKG1* gene that we had previously identified. Based on functional annotation, the genes *TMCO1* on SSC4 and *CKB* on the scaffold represent promising candidate genes for the other two QTL. There were significant interaction effects of the GWAS tag SNPs at those two loci with *PRKAG3* R200Q on RG. In addition, a number of common variants with potentially smaller effects on RG (*P* < 10^−4^) were uncovered by a second conditional GWAS after adjusting for the two causal SNPs, R200Q and g.8283C>A.

**Conclusions:**

We found that the *RN*^–^ allele segregates in the parental lines of our DLY population and strongly influences its meat quality. Our findings also indicate that the genetic basis of RG in DLY can be mainly attributed to two major genes (*PRKAG3* and *PHKG1*), along with many minor genes.

**Electronic supplementary material:**

The online version of this article (10.1186/s12711-019-0488-0) contains supplementary material, which is available to authorized users.

## Background

Pork quality is an economically important trait in the pig industry and its improvement has been one of the major goals in modern pig breeding [1–4]. However, it is difficult to improve pork quality by traditional breeding methods due to its complexity and low heritability [5], and because it can be only observed after slaughter. Therefore, understanding the genetic mechanisms that underlie traits related to meat quality in pigs is very important.

Following the advent of genome-wide association studies (GWAS) and larger-scale consortia meta-analyses of GWAS, about 9900 variants associated with pork quality traits have been identified (http://www.animalgenome.org/cgi-bin/QTLdb/SS/index), which provide valuable insights into the genetic architecture of these traits. However, these GWAS are typically designed to provide a survey of common variants with a minor allele frequency (MAF) higher than 0.05 and, thus, examine only a portion of the genomic landscape of complex traits. Until now, low-frequency (0.01 ≤ MAF < 0.05) and rare (MAF < 0.01) variations that affect pork quality have been more challenging to access. In addition, although GWAS can be used to discover significant association signals for a trait, uncovering the specific genetic variants and pathways that cause these associations is still a major challenge in the post-GWAS era. To date, only a limited number of studies have investigated the molecular mechanism of GWAS hits with fine-mapping [6]. For instance, nearly 8 years and the efforts of many researchers were necessary to unravel the molecular mechanism of the first GWAS-identified obesity gene, *FTO* (*fat mass and obesity*-*associated protein*), via integrative analyses of epigenomic data, allelic activity, motif conservation, gene regulator expression and gene co-expression patterns, and target genome editing verification [7]. Not surprisingly, to date, only a handful of causative genes for meat quality traits have been identified. A recent systems genetic analysis uncovered the role of a splice mutation in the *PHKG1* gene in the regulation of muscle glycogen level, pH, and drip loss in Duroc crosses [8].

After slaughter, muscle glycogen in pigs is converted into lactate by glycolysis and this accumulation of lactate results in a decline of pH during the conversion of muscle to meat. Monin and Sellier [9] suggested that a higher initial muscle glycogen level confers an increased capacity for postmortem glycolysis, or high “glycolytic potential” (GP) that, in turn, enhances the pH decline. Because the rate and extent of postmortem pH decline influence pork quality substantially, measuring GP or postmortem glucose concentrations could be very useful to predict pork quality traits [10]. Furthermore, the level of glycogen is more affected by genetic than by environmental factors, and thus it is more accurate than the measurement of pH.

To our knowledge, *PRKAG3* was the first identified causative gene that is involved in muscle glycogen level and meat quality traits [11]. This gene encodes the AMP-activated protein kinase (AMPK) γ3 subunit. AMPK is an important energy sensor protein kinase that plays a key role in regulating cellular energy metabolism. In Hampshire pigs, several studies have shown that the *PRKAG3* R200Q (p.Arg225Gln) missense mutation is responsible for excess glycogen and low ultimate pH in skeletal muscle, which results in “acid meat”, which has lower water holding capacity and poor processing yield [11–13]. Three other missense mutations in the *PRKAG3* gene, T30N (p.Thr30Asn), G52S (p.Gly52Ser), and I199V (p.Ile199Val), were also suggested to affect meat quality traits in a wider range of commercial pig breeds, e.g. Large White, Duroc, and Landrace [14]. The I199V and neighbouring R200Q mutations are located in a highly conserved region of the cystathionine β-synthase domain, but show opposite effects on meat quality [15].

Several GWAS have been performed on meat quality traits, such as pH and colour, but there have been relatively few studies on the genetic basis of muscle glycogen and lactate. Since the concentrations of these metabolites can be regarded as intermediate phenotypes that affect meat quality, identifying overlapping quantitative trait loci (QTL) for muscle glycogen, pH, and other meat quality traits will facilitate the discovery of candidate genes and the underlying molecular mechanisms.

In China, more than 70% of the pork produced originates from three-way crossbred Duroc × (Large White × Yorkshire) (DLY) pigs, but the high incidence rate of PSE (pale, soft and exudative), DFD (dark, firm and dry), and acid meat incurs consumer dissatisfaction and significant economic loss. Thus, it is important to investigate the genetic architecture of meat quality traits in DLY pigs. Previously, we reported the detection of genetic variants that are significantly associated with multiple meat quality traits but not for GP-related compounds traits [16]. Thus, our objective here was to identify genetic variants and putative causative genes that are associated with muscle glycogen level.

## Methods

### Animal populations

This study involved two cohorts of DLY commercial pigs: (1) a discovery cohort that consisted of 306 males and 304 females raised on a farm of the Jiangxi Guohong Group Co. Ltd in Jiujiang city (Jiangxi Province), where the DLY piglets were produced by crossing ~ 20 adult Duroc boars with ~ 800 L × Y sows; and (2) a replication cohort that comprised 150 DLY pigs, which came from a farm of the Guangdong Wen’s Food Group Co. Ltd in Guangzhou city (Guangdong Province). All experimental individuals were raised under a standardized environment and slaughtered at local commercial abattoirs when they reached a live weight of ~ 110 kg. To constitute these cohorts, 30 to 40 individuals were randomly sampled from groups of 150 or more finishing pigs that were sent for slaughter at the same time. Thus, the cohorts were completely unrelated and the kinship between animals in each cohort was very low.

In addition, 179 Duroc, 192 Landrace, and 190 Yorkshire pigs from various farms in Yongchun country (Fujian Province) and Qingyuan city (Guangdong Province) were also included in the study. These individuals were genotyped for the *PRKAG3* R200Q locus and had no direct relationship with the above-mentioned DLY cohorts.

### Phenotypic analysis

The levels of residual glycogen and glucose (RG) and of lactate in the longissimus muscle (LM) were determined for the two DLY cohorts. The standard equation to calculate the glycolytic potential (GP) of muscle is GP (μmol/g of wet muscle) = 2 × (glycogen + glucose + glucose-6-phosphate) + lactate. However, based on our observations that the level of glucose-6-phosphate is much lower than that of other components in the post-mortem muscle, we ignored it and used the reduced equation to: GP = 2 × RG + lactate. After slaughter, LM samples were collected between the tenth rib and the first lumbar vertebra from the left side of each carcass within 45 min post-mortem. We estimated the levels of RG and lactate in the LM with the muscle glycogen assay (Item No: A043) and the lactic acid assay (A019-2) kits, respectively, which were produced by the Nanjing Jiancheng Bioengineering Institute. In addition, pH, drip loss, and colour were measured on LM at 36 h post-mortem in the discovery cohort, as described previously [16].

### SNP array genotyping and quality control

Pig genomic DNA was isolated from ear tissue by standard phenol/chloroform extraction. To fulfil the requirements for genotyping, all extracted DNA samples were standardized to a final concentration of 50 ng/µl and quality controlled. In total, 610 animals from the Guohong DLY cohort were genotyped for 61,565 SNPs using the IlluminaPorcineSNP60K Beadchip according to the standard manufacturer’s protocol. Quality control procedures were performed by PLINK v1.07 [17]. Briefly, SNPs with a call rate lower than 0.99, a MAF lower than 0.01, and SNPs that deviated from Hardy–Weinberg equilibrium (HWE) (P < 10^−5^) were removed, and individuals for which the call rate was lower than 0.90 and the Mendelian inheritance error rate was higher than 0.05 were also removed from the dataset. After quality control, 39,369 SNPs and all individuals remained for subsequent statistical analyses. The chromosomal positions of SNPs were based on the Sscrofa11.1 pig genome assembly.

Previously, we used this DLY cohort to detect QTL for many meat quality traits except GP-related traits by performing a GWAS that was based on the Sscrofa10.2 reference genome [16]. Recently, the porcine reference genome was updated to the Sscrofa11.1 version with better assembly quality, which improved the sequence alignment for the tested SNPs. Therefore, we also re-analyzed the other meat quality traits with the updated data for GWAS, to help identify potential common genetic loci between GP-related traits and other meat quality traits.

### Genome-wide association analyses for meat quality traits

Associations between each SNP and the studied phenotypic traits were obtained by using a general linear mixed model [18]. The genetic variability and heritability of the traits were estimated in the discovery DLY cohort. Heritability of a trait was estimated by using the *polygenic* function of GenABEL v1.7 [18]. The model used for the GWAS included a random polygenic effect, with a variance–covariance matrix that was proportional to genome-wide identity-by-state:

$$ {\mathbf{y}} = {\mathbf1}\mu + {\mathbf{Xb}} + k{\mathbf{w}} + {\mathbf{Sc}} + {\mathbf{Za}} + {\mathbf{e}}, $$

where ${\mathbf{y}}$ is the vector of phenotypes, $\mu$ is the overall mean, ${\mathbf{b}}$ is the vector of fixed effects, including sex and slaughter batch (N = 18) effects, ${\mathbf{w}}$ is the vector of carcass weight, which was considered as a covariate, ${\mathbf{c}}$ is the SNP substitution effect, ${\mathbf{a}}$ is the vector of polygenic effects with ${\mathbf{a}}\sim N\left( {0, {\mathbf{G}}\upsigma_{{\mathbf{a}}}^{2} } \right)$, where ${\mathbf{G}}$ is the genomic relationship matrix, which was calculated based on the identity-by-state of the SNP on autosomes [19], and $\upsigma_{{\mathbf{a}}}^{2}$ is the polygenetic additive variance; $k$ is the regression coefficient for carcass weight, and ${\mathbf{e}}$ is the vector of residual errors with ${\mathbf{e}}\sim N\left( {0, {\mathbf{I}}\upsigma_{{\mathbf{e}}}^{2} } \right)$, where ${\mathbf{I}}$ is the identity matrix and $\upsigma_{{\mathbf{a}}}^{2}$ is the residual variance. ${\mathbf{X}}$ and ${\mathbf{Z}}$ are the incidence matrices for ${\mathbf{b}}$ and ${\mathbf{a}}$, respectively; ${\mathbf{S}}$ is the incidence vector for ${\mathbf{c}}$. All GWAS for meat quality traits were performed with the GenABEL package in the R environment [18]. Conditional GWAS was conducted by adjusting phenotypes for the top SNP that was identified in the previous round of the scan. Genome-wide and suggestive significance thresholds were set at 0.05/N and 1/N, where N is the number of SNPs tested in the analyses, based on the Bonferroni correction method [20, 21]. The genome-wide and suggestive significance thresholds were 1.27e−6 (0.05/39,369) and 2.54e−5 (1/39,369), respectively, for the DLY population. The proportion of phenotypic variance explained by the top SNPs was estimated by $\left( {{\text{V}}_{\text{reduced}} - {\text{V}}_{\text{full}} } \right)/{\text{V}}_{\text{reduced}}$, where ${\text{V}}_{\text{full}}$ and ${\text{V}}_{\text{reduced}}$ are the residual variances of the models for association analysis with and without the SNP term, respectively.

### Population stratification and estimation of linkage disequilibrium

Population stratification can lead to false positive results in GWAS, thus it was assessed by examining the distribution of the test statistics that were generated from the thousands of association tests and their deviation from the null distribution in quantile–quantile (Q–Q) plots [22], which were constructed using the R software.

We analyzed the patterns of LD blocks for the chromosomal regions that contained multiple significant SNPs clustered around the top SNP. The LD block patterns were built by using the Haploview version 4.2 software with default settings [23]. The LD based on r^2^ was estimated for all pairs of SNPs within the QTL region by using PLINK v1.07 [17].

### Genotyping of variants in the *PRKAG3* and *PHKG1* genes

Because the most significant GWAS top SNP rs326377357 (at 117,255,752 bp) for RG was detected in a region on SSC15 (SSC for *Sus scrofa* chromosome), which is close to the *PRKAG3* gene (at 120.85–120.87 Mb) and overlapped coincidently with other significant loci for RG-related traits (from 114.8 to 121.4 Mb), we attempted to identify the causative variant(s) in this gene. First, a comparative analysis of the complete *PRKAG3* sequence including its promoter [for information on primers (see Additional file 1: Table S1)] was conducted by sequencing DNA from six DLY individuals that showed extreme RG and different genotypes at the GWAS tag SNP rs326377357. Then, the *PRKAG3* R200Q (*RN*^–^) mutation was detected by the PCR–RFLP method, as described previously [24]. In addition, 72 markers, i.e. 30 SNPs in *PRKAG3* that were identified by sequencing and the 42 surrounding SNPs that were identified from swine genome databases, were genotyped for the whole DLY population using a Sequenom Massarray system at Shanghai Genesky Biotechnologies Inc. On SSC3, a splice mutation g.8283C>A in the *PHKG1* gene that may explain the effect of another identified locus affecting RG and meat quality traits was also genotyped, as described by Ma et al. [8].

### Phylogenetic analyses

For the *PRKAG3* gene and the 30-Mb region around *PRKAG3*, the genetic relationships between haplotypes in five commercial breeds were assessed by maximum likelihood (ML) analyses. These analyses involved 52 pigs, including 17 DLY, five Duroc, 10 Landrace, 16 Yorkshire, and 4 Hampshire pigs, among which the 17 DLY and 3 of the Hampshire pigs were heterozygous for the *PRKAG3* R200Q variant, whereas the remaining Hampshire pig was homozygous. Since only 60 K SNPs and the 73 additional markers that were located in the vicinity of *PRKAG*3 were genotyped for the DLY pigs, the same SNPs were extracted from publicly available or our own genome-sequence data for the 35 selected purebred pigs. At the target regions, haplotypes were phased using PHASEBOOK [25]. Maximum-likelihood phylogenetic trees were constructed for the haplotypes using 1000 bootstraps via MEGA v7 [26].

### Single-SNP and haplotype association analysis and SNP–SNP interaction analysis

Association analyses were performed either for individual SNPs within the *PRKAG3* gene (T30N, G52S, I199V, R200Q) or for haplotypes that contained linked markers (T30N, G52S, L53P, 193A, 194L, I199V and R200Q) in this gene. Associations of a SNP (or haplotype) with meat quality traits were examined using the least square means method of the GLM (general linear model) procedure in the R software (version 3.5.0), with a model that included the fixed effects of sex, slaughter batch, and the effect of a SNP (or a haplotype), along with a residual error as a random effect. The SNP–SNP (or QTL–QTL) interaction analysis for three GWAS tag SNPs (*PRKAG3* R200Q, rs81347579 and rs319599168) was performed in R with a model that was identical to that used for SNP association analysis, except that interaction effects between SNPs were included. The significance values for interaction effects of pair-wise and of three SNPs on RG were calculated by two-way and three-way ANOVA, respectively. Plots for the main effects of the three SNPs and their two-way interactions were generated by using the *interaction2wt* function of HH package in the R software.

## Results

### Correlations between muscle glycogen level and meat quality traits

In pigs, glycogen level in the muscle at slaughter influences meat quality. Thus, our aim was to evaluate the impact of variants associated with muscle glycogen level on several meat quality traits. First, we found that RG level in muscle from DLY pigs at 45 min post-mortem was negatively associated with pH 36 h (see Additional file 2: Figure S1) and subjective color score, but was positively associated with drip loss and color L* (*P* < 0.001; Table 1). Moreover, the absolute values of the correlation coefficients between RG and these meat quality traits were all higher than 0.4, which suggest that pigs with a higher RG level produce meat with lower ultimate pH, water holding capacity and color preference. The results were similar for the correlations of GP with these same meat quality traits, except that the correlation coefficients were somewhat lower than for RG level, likely because of the non-significant correlations of lactate level with other meat quality indexes, except with b*.

**Table 1:** Correlation coefficients for levels of RG and lactate, GP, and six other meat quality traits in the DLY pigs

Traits	RG	Lactate	GP
Lactate	− 0.03 ns		
GP	0.57***	0.80***	
pH 36 h	− 0.40***	0.00 ns	− 0.24***
Drip loss	0.46***	0.06 ns	0.32***
Color score	− 0.41***	− 0.07 ns	− 0.30***
Color L*	0.42***	0.08 ns	0.32***
Color a*	0.25***	− 0.01 ns	0.14*
Color b*	0.46***	0.09*	0.34***

*ns* not significant

* *P* < 0.05; ** *P* < 0.01; *** *P* < 0.001

### Genetic variability and heritability estimates for three GP-related traits in DLY pigs

Descriptive statistics and heritability estimates for GP, and levels of RG and lactate are in Table 2. For all three traits, coefficients of variation (CV) were higher than 37%, which indicates that they show considerable variation in DLY crossbred pigs. Heritability estimates for GP and RG were moderately high (0.27 and 0.40, respectively), but much lower for lactate (0.04). In a previous study, we reported moderately high heritabilities (ranging from 0.34 to 0.42) for several meat quality traits associated with RG level in this DLY population, such as pH 36 h, drip loss, and meat colour indicators [16].

**Table 2:** Descriptive statistics and heritability estimates for glycolytic potential (GP), and levels of residual glycogen and glucose (RG) and lactate in 610 DLY pigs

Traits	Maximum (μmol/g)	Minimum (μmol/g)	Mean ± SD (μmol/g)	CV (%)	Heritability
GP	236.24	25.67	84.38 ± 31.51	37.34	0.27
RG	84.15	0.44	9.89 ± 9.43	95.32	0.40
Lactate	187.82	15.78	64.58 ± 25.63	39.70	0.04

### No evidence of population stratification

For the GWAS, an average inflation factor (λ) of 1.003 was found for GP, RG, lactate, and other meat quality traits in the DLY population, which indicates no evidence of population stratification. The Q–Q plots of the test statistics for each separate GWAS are in Additional file 3: Figure S2.

### Detection of three genomic loci that affect RG level in the initial GWAS

**Fig. 1:**
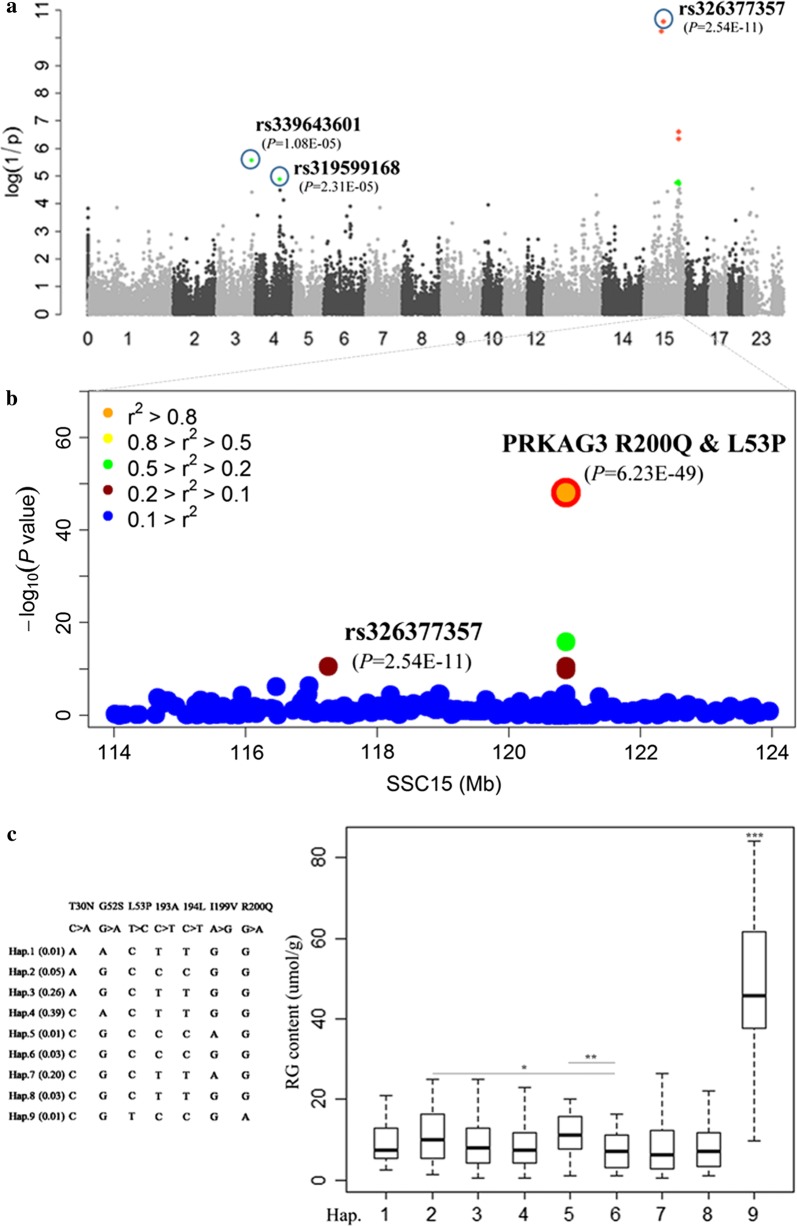
Pinpointing *PRKAG3* R200Q as underlying a major QTL for residual glycogen level (RG) in muscle on SSC15. **a** A Manhattan plot of the initial GWAS for RG level with 60 K SNP data. The red and green dots represent the SNPs that reached genome-wide significance (*P* < 1.27e−6) and chromosome-wide significance level (*P* < 2.54e−5). **b** Regional association plot for RG level in the region between 114 and 124 Mb on SSC15 where 73 additional markers were incorporated. Two completely-linked and top SNPs were revealed: *PRKAG3* R200Q and L53P (highlighted by red and orange dots, respectively), which were far more significant than the original top SNP rs326377357. The levels of linkage disequilibrium (LD) between the *PRKAG3* R200Q and its surrounding SNPs are indicated by different colors. **c** Effects of nine haplotypes that include seven SNPs (T30N, G52S, L53P, 193A, 194L, I199V and R200Q) in *PRKAG3* on RG level. Only the ninth haplotype that includes the 200Q and 53L alleles could cause a much higher RG level compared to the other eight haplotypes (*P *< 0.001)

Our initial GWAS on the DLY population demonstrated that 10 SNPs, representing three genomic loci, were significantly associated with RG (Table 3 and Fig. 1a). Unsurprisingly, for GP and lactate level, which had comparatively lower estimates of heritability (Table 2), no significant SNPs were identified. On SSC3, the top SNP rs339643601 (at 123.07 Mb) for RG level was located in a gene desert region, 344 kb from the nearest gene, *LRAD1*, which encodes the LRAT domain containing 1 protein. On SSC4, the top SNP rs319599168 (at 84.98 Mb) is an intronic variant in the *transmembrane and coiled*-*coil domains 1* (*TMCO1*) gene. The most significant SNP (rs326377357 at 117.26 Mb; *P *= 2.54e−11) for RG level was on SSC15, rs326377357 (at 117.26 Mb; *P *= 2.54e−11), which was surrounded by seven other significant SNPs. Intriguingly, the region between 114.8 and 121.4 Mb on SSC15 also contained many SNPs that were significantly associated with most other RG-related traits, e.g. pH 36 h, drip loss, color score, and three color parameters, L*, a* and b* (Table 3). Moreover, this region harbors the *PRKAG3* gene (at 120.86 Mb), which has been shown to impact these meat quality traits in Hampshire pigs. Thus, *PRKAG3* is the most likely causative gene for the SSC15 QTL based on its position and functional annotations.

**Table 3:** Top GWAS SNPs for RG level and for six other meat quality traits within the *PRKAG3* locus in the DLY pigs

Traits	Peak SNP	N^a^	Chr	Pos (bp)^b^	Nearest or candidate gene^c^	MAF^d^	Effects^e^	*P*-value^f^
RG	rs339643601	1	3	123,067,585	*LRATD1*	0.09	0.77	1.08 × 10^−5^
RG	rs319599168	1	4	84,976,996	*TMCO1*	0.06	3.00	2.31 × 10^−5^
RG	rs326377357	8	15	117,255,752	*PRKAG3*	0.21	10.55	2.54 × 10^−11$^
pH 36 h	rs81454395	1	15	114,816,163	*PRKAG3*	0.41	0.06	3.99 × 10^−5^
Color score	rs80853255	1	15	115,312,329	*PRKAG3*	0.44	0.15	1.84 × 10^−5^
Color L*	rs80853255	1	15	115,312,329	*PRKAG3*	0.44	0.82	3.64 × 10^−4^
Color a*	rs80925998	1	15	121,364,252	*PRKAG3*	0.37	0.25	1.44 × 10^−5^
Color b*	rs80925998	1	15	121,364,252	*PRKAG3*	0.37	0.32	4.11 × 10^−5^
Drip loss	rs80925998	2	15	121,364,252	*PRKAG3*	0.37	0.42	9.92 × 10^−6^

^a^Number of SNPs that surpassed the suggestive significance level within the QTL regions

^b^Positions of the most significant SNP according to the *Sus Scrofa* Build 11.1 assembly

^c^Annotated gene nearest to the most significant SNP or involved in energy homeostasis (candidate gene shown in italics); gene names starting with ENSSSCG follow the Ensembl nomenclature while other gene symbols follow the HUGO nomenclature

^d^Minor allele frequency

^e^Additive effects

^f^Genome-wide significant association is indicated with a $ symbol

### Identification of the *PRKAG3* R200Q variant that is responsible for the major SSC15 QTL

To search for putative causal variants in the *PRKAG3* gene, we sequenced it using DNA samples from six DLY animals that comprised the three different genotypes at the top SNP rs326377357 (2 individuals per genotype), among which the alternate homozygous individuals had high versus low RG levels. We detected 53 SNPs within the *PRKAG3* gene (see Additional file 4: Table S2). Surprisingly, the R200Q substitution, at which the 200Q allele was previously detected in the Hampshire breed but not in other breeds [11, 14], did segregate in our samples.

It is known that the *PRKAG3* R200Q mutation causes a ~ 70% increase in muscle glycogen level in *RN*^–^ (*RN*^–^/rn^+^ or *RN*^–^/*RN*^–^) Hampshire pigs [11], thus we examined the effects of this mutation on meat quality traits in the DLY pigs, which included only two genotypes (*RQ* and *RR*). In fact, highly significant differences were observed between these two genotypes for all tested traits, including RG level, GP, pH 36 h, drip loss, and three color parameters of meat (L*, a*, b*) (Table 4). Notably, *RQ* carrier individuals had a nearly fivefold higher RG level (*P *= 5.75e−49) and a twofold greater drip loss (*P* = 2.93e−12) than the wild-type *RR* animals, which confirms that the dominant R200Q (*RN*^–^) mutation has a strong influence on meat quality of DLY pigs.

**Table 4:** Effects of the *PRKAG3* R200Q mutation on meat quality traits

Traits	RR (n = 590)	RQ (n = 17)	Effect	*P*-value
RG	8.88	43.98	17.56	5.75 × 10^−49^
GP	82.58	147.14	32.28	8.87 × 10^−16^
pH 36 h	5.50	5.30	0.10	3.61 × 10^−6^
Drip loss	3.30	6.49	1.60	2.93 × 10^−12^
Color score	3.07	2.35	0.36	1.11 × 10^−8^
Color L*	46.92	51.68	2.38	1.96 × 10^−12^
Color a*	1.48	2.70	0.61	1.06 × 10^−7^
Color b*	5.62	7.87	1.13	9.46 × 10^−13^

**Fig. 2:**
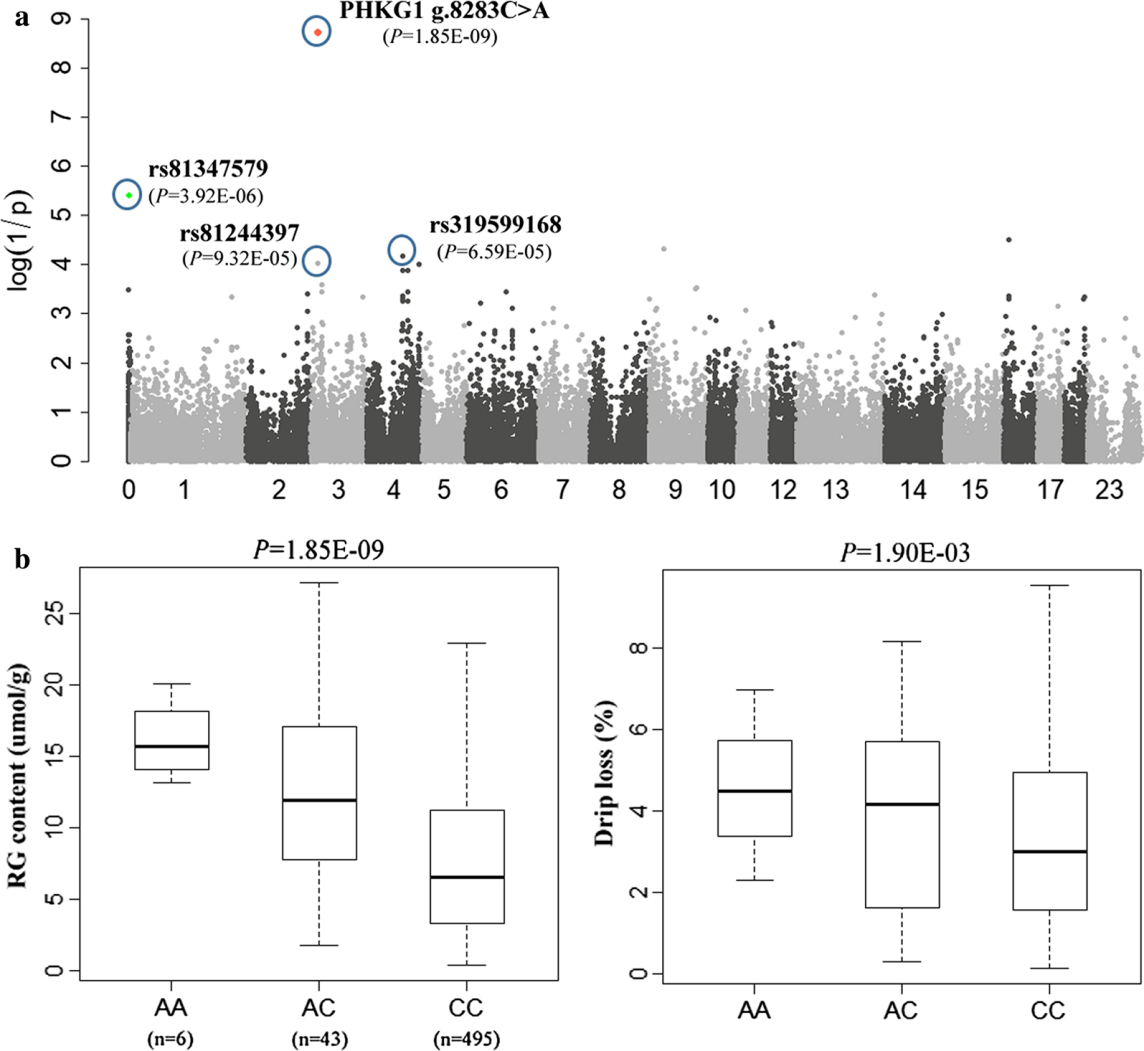
Pinpointing *PHKG1*g.8283C>A as underlying another major QTL for RG on SSC3. **a** Manhattans plot of the GWAS for RG level after fitting the *PRKAG3* R200Q SNP in the model. **b** Difference in average of RG level and drip loss between the three genotypes of the *PHKG1*g.8283C>A SNP

Some studies have suggested that, in addition to the R200Q variant, other variants and/or genes in the region flanking *PRKAG3* may have significant effects on meat quality in pigs [14, 27–29]. Therefore, to investigate the genomic architecture of this region in detail and to determine whether other variants in this region contributed to the QTL effect, we extended the analysis to other variants in *PRKAG3* and neighboring genes. Based on the genotyping data for the six DLY pigs mentioned above, we identified nine haplotypes in the *PRKAG3* region (see Additional file 5: Figure S3). Accordingly, we selected 31 SNPs out of the 53 identified SNPs in *PRKAG3* (including all 8 missense mutations and discarding 22 intronic SNPs) for further analysis. In addition to the R200Q variant, we genotyped 72 SNPs that were located in 11 genes (see Additional file 6: Table S3) in the complete set of DLY pigs and re-ran the GWA analysis. Results showed that the *PRKAG3* R200Q and L53P mutations, which were in complete LD with each other, showed many more (> 10^3^) significant associations with the meat quality traits studied here than any other tested SNP (Fig. 1b) and (see Additional file 7: Figure S4). In addition, after fitting the R200Q substitution in the model (Fig. 2a) or removing all 17 heterozygous *RQ* individuals from the data (data not shown), the association between the *PRKAG3* locus and the meat quality traits was no longer significant, even at a significance level of 0.01. These results indicate that the R200Q substitution accounts for most, if not all, of the effects of the QTL on SSC15.

Three additional missense mutations (T30N, G52S and I199V) in *PRKAG3* that have been reported to influence muscle glycogen level [14], also segregated in the DLY population with a MAF higher than 0.22. Our GWAS showed that these three mutations were significantly (*P* < 0.05) associated with several meat quality traits (Table 5), but none reached the significance level of the original GWAS tag SNP rs326377357 (*P* = 2.54e−11) and R200Q (*P* = 6.23e−49). Moreover, we found that, in the DLY replication cohort, the R200Q and L53P mutations also had a stronger effect on RG level than the G52S and I199V mutations (see Additional file 8: Table S4). In addition, we discovered nine haplotypes that comprised seven closely linked markers in *PRKAG3,* including T30N, G52S, L53P, 193A, 194L, I199V and R200Q. Among these, only one haplotype “30T(C)-52G(G)-53L(T)-193A(C)-194L(C)-199V(G)-200Q(A)” (denoted as *Hap9*) contained the 200Q alleles, and its effects on RG level, pH 36 h, and drip loss differed significantly from those of the other haplotypes (*P *< 0.001; Fig. 1c). Taken together, these results provide additional support for the *PRKAG3* R200Q mutation as the major contributor to the GWAS signals identified in the region.

**Table 5:** Effects of the three missense mutations in *PRKAG3* on meat quality traits in DLY pigs

Traits	T30N*				G52S				I199V			
	TT	TN	NN	*P*-value	GG	GS	SS	*P*-value	II	IV	VV	*P*-value
	(N = 298)	(N = 214)	(N = 88)		(N = 220)	(N = 291)	(N = 97)		(N = 37)	(N = 183)	(N = 366)	
RG	9.52	10.42	10.30	0.51	10.97^a^	9.40^ab^	8.76^b^	0.05	8.82	9.18	10.51	0.08
Lactate	61.64^a^	66.15^ab^	69.38^b^	0.05	65.69	63.99	63.78	0.87	55.93^a^	63.57^ab^	65.88^b^	0.04
GP	80.69	86.99	89.99	0.16	87.90	82.81	81.31	0.25	73.47^a^	81.93^ab^	86.99^b^	0.01
pH 36 h	5.51	5.48	5.47	0.19	5.47	5.52	5.52	0.89	5.52^ab^	5.52^a^	5.49^b^	0.01
Drip loss	3.13^a^	3.58^b^	3.68^bc^	0.04	3.69	3.20	3.01	0.14	3.10^a^	3.17^ab^	3.47^b^	0.05
Color score	3.07	3.00	3.06	0.70	3.03	3.07	3.05	0.46	3.05	3.09	3.02	0.08
Color L*	46.80	47.42	47.04	0.74	47.25	46.90	46.87	0.95	47.24	46.86	47.17	0.29
Color a*	1.43^a^	1.54^ab^	1.76^b^	0.007	1.67^a^	1.42^b^	1.40^b^	0.04	1.44^ab^	1.37^a^	1.58^b^	0.008
Color b*	5.50^a^	5.86^b^	6.00^bc^	0.01	5.89	5.53	5.66	0.17	5.58^ab^	5.43^a^	5.86^b^	0.002

* Means of different genotypes at a given SNP in rows with different superscript letters are significantly (*P* < 0.05) different from each other

**Fig. 3:**
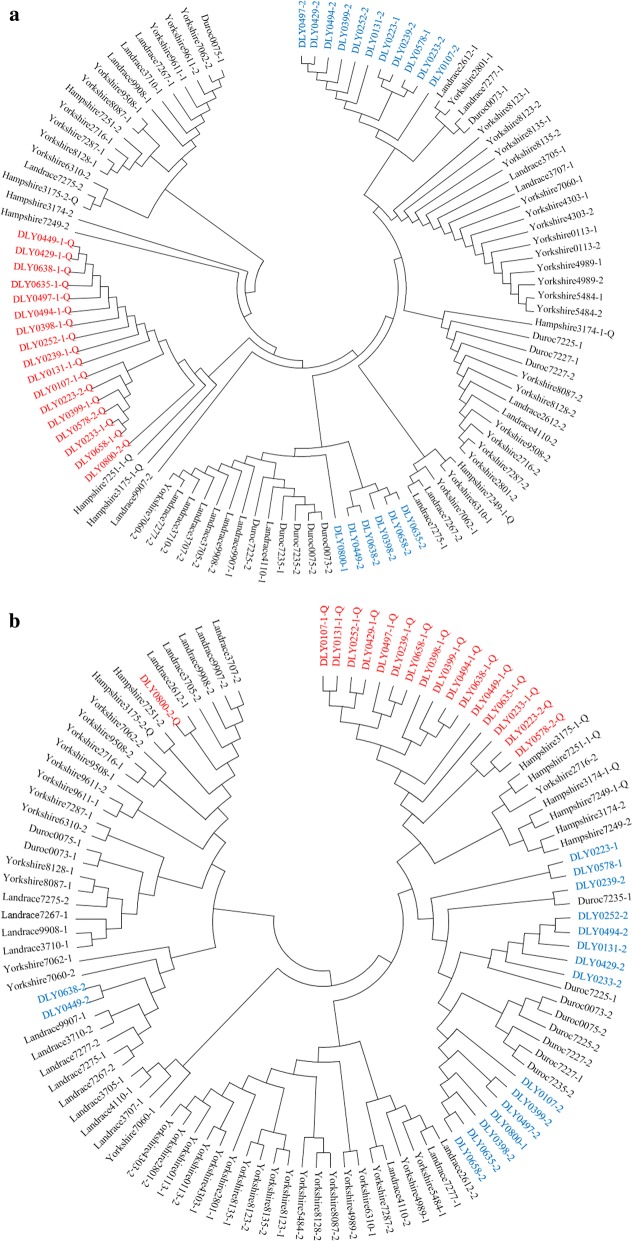
The haplotypes that harbor the *PRKAG3* 200Q allele in DLY pigs may have been introgressed from the Hampshire breed. **a** Maximum-likelihood (ML) tree for haplotypes at the *PRKAG3* gene. **b** ML tree for haplotypes at the 30-Mb region centered on *PRKAG3*. Two haplotypes from each individual are denoted by “1” or “2” at the end of its name. The letter “Q” after the haplotype number indicates the haplotypes that carry 200Q. The Q- and R-types of haplotypes in the 17 heterozygous DLY pigs are highlighted by red and blue, respectively

Notably, the *Hap9* haplotype detected in the DLY population is exactly the same as the *RN*^–^ haplotype that was found exclusively in the Hampshire breed by Milan et al. [11], which suggests that the *RN*^–^ allele has probably been introgressed from Hampshire pigs into the parental lines of the DLY pigs. To validate this, we constructed two maximum likelihood (ML) trees for haplotypes for the *PRKAG3* region (120.84–120.86 Mb) and for a more extensive region around *PRKAG3* (105.83–135.83 Mb), using the genotyping data for carriers of the *RN*^–^ allele in DLY and Hampshire, and non-carriers of the *RN*^–^ allele in Duroc, Landrace, and Yorkshire pigs. As expected, both ML trees showed that almost all 200Q-haplotypes from DLY pigs clustered with the *RN*^–^ haplotype of the Hampshire breed (Fig. 3a, b). We then tried to determine in which of the parental lines of the DLY pigs the *RN*^−^ allele was segregating. Since only the sires (Duroc boars) of the DLY discovery cohort were sampled for DNA, they were the only ones genotyped for the *RN*^−^ mutation. We found that the 200R allele was fixed in these individuals, which suggests that the DLY pigs inherited this allele only from their mothers (L × Y sows). This is consistent with the results of the phylogenetic analysis that showed that the majority of the 200R (rn^+^)-haplotypes for the 30-Mb region around *PRKAG3* in the DLY pigs were more closely related to the Duroc’s haplotypes than to Landrace and Yorkshire haplotypes (Fig. 3b). Since the *RN*^–^ allele was apparently introduced in the ancestors (Landrace or Yorkshire) of our DLY population several or many generations ago, we assumed that the DLY heterozygous individuals contained recombinant chromosomes that combined the classical *RN*^−^ haplotype with haplotype fragments that originated from the Landrace or Yorkshire lines. This hypothesis was confirmed by the detection of a ~ 2.38-Mb (between 117.70 and 120.08 Mb) haplotype fragment of Yorkshire origin that was located just upstream of the *RN*^−^ haplotype in three of the heterozygous DLY individuals (see Additional file 9: Table S5), suggesting that their *RN*^−^ alleles originated from Yorkshire. In addition, the ML tree analysis showed that one DLY 200Q-haplotype (DLY0800-2-Q) clustered with Landrace haplotypes (in particular, with Landrace2612-1) (Fig. 3b), and indeed these shared the same haplotype fragment upstream of *PRKAG3* (see Additional file 9: Table S5), which indicates that the *RN*^–^ allele of this DLY pig likely derived from Landrace. Based on these results, we infer that the *RN*^−^ allele may be segregating in both the Yorkshire and Landrace parental lines.

To further verify the existence of the *RN*^-^ allele in purebred Landrace, Yorkshire, and Duroc pigs, we genotyped it in about 190 individuals per breed from three farms, which were unrelated to the current DLY population. We did not detect the *RN*^-^ allele in the Landrace and Yorkshire pigs, but unexpectedly we identified it in the Duroc boars from a farm in Qingyuan city at a frequency of 0.14. Taken together, these results indicate that the *RN*^−^ allele was introgressed into the three commercial pig breeds from Hampshire pigs.

### Identification of the *PHKG1*g.8283C>A variant that is responsible for the SSC3 QTL effect

**Fig. 4:**
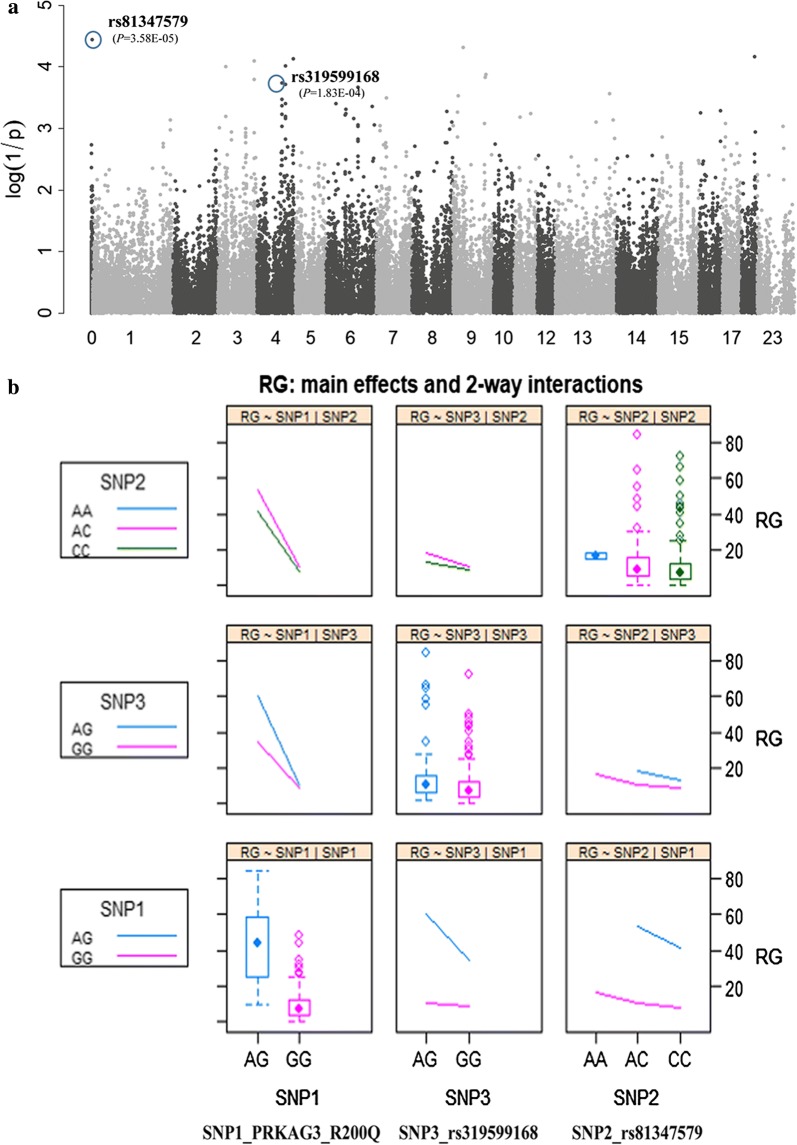
Uncovering other potential loci for RG level and evaluating their effects. **a** Manhattans plot of the conditional GWAS when adjusting for two pinpointed causal variants (*PRKAG3* R200Q and *PHKG1*g.8283C>A) in the model. One top SNP rs81347579 that is currently assigned to “chromosome unknown” and another SNP rs319599168 on SSC4 that were identified in the earlier GWAS (Figs. 1a, 2a) are still significantly associated with RG level. **b** The main-effects of the three top SNPs (*PRKAG3* R200Q, rs81347579 and rs319599168) and their two-way interaction effects on RG level

Previously, we reported a causal relationship between the *PHKG1*g.8283C>A variant and muscle glycogen level and meat quality traits in different Duroc crossbreds [8], including 140 samples from the current discovery cohort because, at the time, 60K genotype data and GP phenotype data were not available for all pigs. Interestingly, results of the GWAS when conditioning on *PRKAG3* R200Q or by excluding the 17 heterozygous individuals at R200Q, showed that, on SSC3, the original top SNP rs339643601 (at 123.07 Mb) near *LRATD1* disappeared, while rs81244397 (at 15.95 Mb; *P* = 9.32e−5) became a prominent SNP (Fig. 2a), which is located near the *PHKG1* gene (at 16.82 Mb). Thus, we included *PHKG1*g.8283C>A in the conditional GWAS to evaluate its potential contribution to the QTL signal. As expected, this previously detected causal variant showed a highly significant association with RG level (*P* = 1.85e−9) and drip loss (*P* = 1.90e−3), and its minor allele “A” (MAF = 0.049) increased RG level and drip loss, with additive effects of 5.82 μmol/g and 0.67%, respectively (Fig. 2b). Furthermore, when SNP g.8283C>A was included in the model as a fixed effect, all the tested SNPs in this QTL region were no longer significant (Fig. 4a).

### Discovery of two GWAS tag SNPs for RG level that interact with *PRKAG3* R200Q

In addition to the *PHKG1* locus, we also found a novel significant SNP rs81347579. This is located on an unassembled scaffold AEMK02000452.1 in the 11.1 assembly, but we were able to deduce that it is very likely residing on SSC7; see footnote of Table 6). This analysis also identified another suggestive SNP rs319599168 on SSC4 (which was previously identified in the initial GWAS) by re-association analyses when adjusting for the *PRKAG3* R200Q SNP in the model (Fig. 2a) or excluding all carriers of the 200Q-allele from the data. Furthermore, even when both *PRKAG3* R200Q and *PHKG1*g.8283C>A were included as fixed effects in the statistic model, rs81347579 and rs319599168 still had significant (*P* < 1.83e−4) associations with RG level (Fig. 4a). Taken together, these results imply that these signals likely represent independent QTL, rather than false positive signals caused by the *PRKAG3* and *PHKG1* variants. Both the rs81347579 and rs319599168 SNPs, had “*A*” as minor allele in the DLY pigs, with frequencies of 13.5 and 5.7%, respectively, and increased RG level by 2.50 ± 0.27 and 2.10 ± 0.26 μmol/g, respectively, based on differences in average RG levels between heterozygotes and homozygotes for the major allele (Fig. 4b), which subsequently caused a reduction in pH 36 h by 0.032 and 0.026, respectively.

**Table 6:** Multiple SNPs associated with RG level at a significance threshold of *P* < 1e−4, detected by the GWAS conditioning on both *PRKAG3* R200Q and *PHKG1*g.8283C>A variants

Top SNP	Chr	Pos (bp)	Nearest or candidate genes^a^	MAF^b^	Effects^c^	*P*-value	Var (%)^d^
**rs81347579**	Unknown^e^	2,127,646^e^	*CKB*	0.14	3.79	3.58E−05	2.37
**rs324148419**	9	35,634,517	*GRIA4*	0.21	2.84	4.76E−05	2.84
**rs81270133**	18	45,962,926	*ENSSCG0000003394* ^f^	0.34	1.74	6.90E−05	1.09
**rs81475445**	4	124,944,340	*BTBD8*	0.28	2.03	7.35E−05	1.58
**rs81240897**	3	123,200,355	*LRATD1*	0.38	3.03	8.01E−05	2.23
**rs80965652**	4	96,151,597	*S100A6*	0.45	1.99	9.62E−05	2.39
**rs81379369**	3	27,217,537	*XYLT1*	0.37	1.27	9.99E−05	0.91

^a^Annotated genes that located nearest to the most significant SNP or involved in energy homeostasis (candidate gene shown in bold); gene names starting with ENSSSCG follow the Ensembl nomenclature while other gene symbols follow the HUGO nomenclature

^b^Minor allele frequency

^c^Additive effects

^d^Phenotypic variance explained by the top SNPs

^e^Position of SNP rs81347579 on the AEMK02000452 scaffold, which is not assigned to any chromosome, a human-pig comparative map and linkage disequilibrium mapping (data not shown) indicate that it is likely located at ~ 120 Mb (near the telomere) on SSC7

^f^Encodes a lincRNA

Intriguingly, the tag SNPs rs81347579 and rs319599168 had significance levels before and after adjusting for *PRKAG3* R200Q that varied to a certain extent (Figs. 1a, 2a), which suggests that they may interact with R200Q. Indeed, Fig. 4b shows a very significant interaction (*P* = 3.34e−10) between SNP rs319599168 and *PRKGA3* R200Q, because the difference in mean RG level between the *AG* and *GG* genotypes at rs319599168 (or R200Q) was much larger in heterozygous individuals at R200Q (or rs319599168) than in homozygous individuals at the same locus. The interaction between rs81347579 and R200Q was also significant (*P* = 0.02), but the interaction between rs81347579 and rs319599168 was not (*P* = 0.20) (Fig. 4b). In addition, a three-way ANOVA also showed significance of the main effects of the three SNPs (*P* < 2e−16, 2.30e−5 and 4.17e−4 for R200Q, rs81347579 and rs319599168, respectively) and of the above-mentioned two-SNP interactions (*P* < 1.97e−9 and 0.033 for R200Q-rs319599168 and R200Q-rs81347579, respectively), as well as of the three-SNP interaction (*P* = 5.63e−6).

### Detection of potential loci that influence RG level

In humans, GWAS that involved tens of thousands of individuals and millions of SNPs revealed significant and suggestive association signals for a complex phenotype at *P*<5e−8 and *P* < 1e−5, respectively [30]. In our study, we applied Bonferroni corrected *P* value thresholds (1.27e−6 and 2.54e−5) in the initial GWAS and the first conditional GWAS. However, for the second conditional GWAS, after controlling for the two causal variants (*PRKAG3* R200Q and *PHKG1*g.8283C>A), we preferred to consider *P* < 1e−4 as the suggestive threshold for the detection of additional QTL for RG level. Based on this *P* value, we identified seven chromosomal regions that harbored suggestive SNPs with a *P* < 1e−4, including one previously identified SNP, rs81347579, close to the *CKB* gene. Notably, nearby the *LRATD1* gene on SSC3, the top SNP, rs81240897 (Table 6), which was detected by the conditional GWAS, was found to be the second strongest signal identified in the initial GWAS that previously revealed rs339643601 as the most significant SNP (Table 3). Although these two GWAS tag SNPs are only 133 kb apart, they were in low LD (r^2^ = 0.17) and had markedly different MAF, which suggests that they may represent distinct QTL. Those suggestive SNPs detected by the conditional GWAS individually explained 0.91 to 2.84% of the phenotypic variance, and their MAF were in the range of 0.14 to 0.45 (Table 6).

## Discussion

### Relationships of GP-related traits with other meat quality traits

Scheffler et al. [31] reported that in “normal” pigs that do not carry the 200Q allele, GP is weakly associated with pH24 h (r^2^ = 0.00, *P* = 0.89). In contrast, we found a slightly stronger and significant association between GP and pH 36 h in all the DLY pigs (r = − 0.24, *P* < 0.001) as well as in DLY pigs that do not carry the 200Q mutation (r = − 0.21, *P* < 0.001). This discrepancy may in part be due to the effect of other *PRKAG3* mutations or other genes in our population. Importantly, our results showed that RG level at an early stage postmortem is more likely to impact meat quality than GP level, because its associations with pH 36 h, drip loss, and meat color parameters were all significant at *P* < 0.001 and at a higher level than the associations of GP level with those same meat quality characteristics (Table 1). Thus, genetic loci that have a greater effect on muscle glycogen level are more likely to affect related meat quality traits (e.g. pH and drip loss).

### Candidate genes

Besides the known causative genes *PRKAG3* and *PHKG1*, two novel candidate genes, *LRATD1* and *TMCO1* on SSC3 and SSC4, respectively, were identified by the initial GWAS (Table 3). An earlier study indicated that up-regulation of *LRATD1* (previously called *FAM84A*) plays a critical role in the progression of colon cancer [32], but its biological function is still not well known. The *TMCO1* gene encodes an endoplasmic reticulum transmembrane protein that forms a functional calcium-selective channel that prevents Ca^2+^ stores from overfilling [33]. The top SNP detected by the GWAS, rs319599168 (g.84976996C>T), is located in an intron of this gene. Interestingly, another calcium channel gene, *RYR1*, which encodes a ryanodine receptor that is present in skeletal muscle and mediates the release of Ca^2+^ from the sarcoplasmic reticulum into the cytoplasm, also contains mutations that cause porcine malignant hyperthermia susceptibility and pale, soft, and exudative meat [34]. This is in agreement with the assumption that causative genes for a trait are functionally closely related [35]. In addition, the most significant SNP identified by conditional GWAS, rs81347579, was mapped 57 kb downstream of the *CKB* gene (Table 6), which encodes creatine kinase that reversibly catalyzes the transfer of phosphate between ATP and various phosphogens (e.g. creatine phosphate). Since the creatine kinase reaction influences ATP and ADP levels, which are critical to glycogen storage in live pigs and to the rate of anaerobic glycolysis and pH decline in postmortem muscle [36], the *CKB* gene is a promising candidate for RG level. Nevertheless, the causality between these candidate genes and RG level needs to be verified by future functional experiments.

It should be noted that screening and prioritizing positional candidate genes depend largely on the precision of QTL mapping and knowledge of their function. Precision of QTL mapping is mainly determined by the extent of LD between markers and a causal variant, which is, however, usually insufficient if the causal variant has a low MAF (< 0.05). The frequency of the *PRKAG3* 200Q mutant allele in DLY pigs was only ~ 1.4%. Retrospective analysis indicated that the top SNP for RG level identified by the initial GWAS, rs326377357 (located at 3.60 Mb from R200Q), was indeed in stronger LD with R200Q (r^2^ = 0.053) than other SNPs that passed quality control. However, when the number of typed SNPs within the candidate region increased, several new SNPs were identified, less than 1.4 kb away from R200Q, which were more tightly linked with R200Q and more significantly associated with RG level than rs326377357 (Fig. 1b). This result supports the precision of our QTL mapping and the hypothesis that increasing marker density is useful to refine QTL location and identify candidate genes, especially for low-frequency QTL.

### Evaluation of the effects of *PRKAG3* R200Q on pork quality in DLY pigs

Our results demonstrated that the 200Q (*RN*^−^) allele increased RG level and GP by approximately fourfold and 87%, and markedly decreased pH 36 h and water-holding capacity (Table 4). Although this marked effect of R200Q on these traits seems comparable in DLY and Hampshire pigs [11], there is some discrepancy. In the Hampshire breed, animals with an RG level higher than 40 μmol/g or a GP value higher than 180 μmol/g can be classified as *RN*^−^/rn^+^ or *RN*^−^/*RN*^−^ with high accuracy (> 95%). However, this simple criterion to infer the genotype at R200Q may not be suitable for DLY pigs, because only 65% (11 out of 17) of the *RN*^−^ carriers in the DLY population met these criteria, while the other seven DLY individuals that had such RG levels and GP values were homozygous for the wild-type genotype (rn^+^/rn^+^). This discrepancy may be due to distinct genetic backgrounds, genetic heterogeneity and/or interactions of R200Q with other loci.

### Introgression of the *RN*^−^ allele from Hampshire pigs to the parental breeds of the DLY crosses

The Hampshire origin of the 200Q (*RN*^−^) allele has been confirmed, since it was shown to be present in a very high percentage of Hampshire pigs, but not in other breeds [14]. However, we demonstrated that not only the 200Q allele but also a specific haplotype that was exclusively found in *RN*^−^ Hampshire pigs was present in DLY pigs. Based on the finding that all Duroc boars that contributed to the DLY population analyzed are normal (rn^+^/rn^+^ genotype), and based on results of the evolutionary tree analysis and the haplotype-sharing analysis of five commercial breeds, we deduced that the 200Q allele was introgressed from the Hampshire breed to the purebred parental lines, including both Landrace and Yorkshire. Surprisingly, we did not identify a Landrace or Yorkshire pig that carries *RN*^−^ allele, probably because of its low frequency, but we did identify carriers of this allele in a Duroc line. Thus, our study provides evidence for the first time that the *RN*^−^ allele may already be segregating in all parental breeds of DLY.

How and when the *RN*^−^ allele was introgressed from the Hampshire breed into the other commercial breeds is unclear. However, as far as we know, the ancestors used to reproduce Duroc, Landrace, or Yorkshire purebred pigs were usually imported from abroad, and none of the breeding programs involved crossbreeding with Hampshire pigs in China. Therefore, we cannot not rule out that some Duroc, Landrace, or Yorkshire pig lines were infused by Hampshire related animals before they were imported into China. Considering the negative effect of the *RN*^−^ allele on meat quality, it is important to eliminate it from the populations in which it segregates.

### Evaluation of the impact of other missense variants in the *PRKAG3* gene

In addition to the R200Q mutation, other mutations in *PRKAG3* may influence meat quality traits, such as the I199V substitution. Ciobanu et al. [14] suggested that this mutation has opposite effects on RG level, GP, pH, and color scores, compared with 200Q. In agreement with that, our results confirmed that the 199I allele was associated with lower glycogen level, higher pH 36 h, and lower drip loss (Table 5). Nevertheless, the effect of the I199V mutation, as well as that of G52S and T30, is not sufficient to account for the QTL effect that we observed in the DLY pigs, which, in contrast, was almost completely explained by the R200Q substitution.

### Interaction between candidate genes

Our analyses identified interactions between the *PRKAG3*-*TMCO1* (R200Q × rs319599168) and *PRKAG3*-*CKB* (R200Q × rs81347579) genes to be significantly associated with RG level, which confers an increased risk for the RN^−^ phenotype. Indeed, we found that the additive effect of the minor allele (*A*) at rs319599168 explained only ~ 3.0 μmol/g (Table 3), while it could result in an approximate doubling (from 34.9 to 60.6 μmol/g) of the RG level in carriers of the 200Q allele (Fig. 4b). More importantly, the GG (rs319599168) × GG (R200Q) combination haplotype seems to be most favorable for pork quality. Further validation of the effects of these gene interactions in other pig populations and investigation of their functional relevance are necessary.

### Major and minor QTL for RG level

In this study, we detected two major QTL (*PRKAG3* R200Q and *PHKG1*g.8283C>A) and at least eight minor QTL (including 7 in Tables 1, 6 adjacent to *TMCO1* on SSC4) for RG level. The former two loci belong to low-frequency variants in the DLY population and together explained nearly 70% of the phenotypic variation in RG level in this population. By contrast, the effect of the minor and common QTL variants (MAF > 5%) was only 3 to 10% of the effect of *PRKAG3* R200Q on RG level and each of them explained 1 to 3% of the phenotypic variance. These results suggest that, in DLY pigs, muscle glycogen level and meat quality traits are regulated by a limited number of major genes and many other minor genes.

## Conclusions

We detected ten chromosomal regions that were significantly associated with RG level in DLY pigs. For the two large-effect QTL, the *PRKAG3* R200Q and *PHKG1*g.8283C>A variants were pinpointed to cause excess glycogen level and result in inferior meat quality traits in DLY pigs. Thus, it is important to eliminate the unfavorable alleles in the Duroc or other parental breeds of DLY, especially the 200Q allele. In addition, two promising candidate genes, *TMCO1* and *CKB*, which play a role in calcium homeostasis and energy homeostasis, respectively, were identified, but their functional validation needs to be investigated further. Interactions of three SNPs, R200Q in *PRKAG3*, rs319599168 in *TMCO1*, and rs81347579 nearby *CKB*, were significant. Our findings provide new insight into the genetic architecture of RG level, GP, and meat quality traits in commercial pig lines and suggest potential candidate loci for pork quality improvement.

## Additional files

**Additional file 1: Table S1.** Primers used for sequencing the *PRKAG3* gene. Fifteen pairs of primers were used to sequence the porcine *PRKAG3* gene and identify variants.

**Additional file 2: Figure S1.** Correlation coefficients between RG level and pH 36 h. The x-axis and y-axis represent RG level and pH 36 h, respectively. Compared with most of the wild-type homozygotes, 17 individuals heterozygous at R200Q tended to have a higher RG level and lower pH 36 h, and six of them were also heterozygous at the GWAS tag SNP rs319599168.

**Additional file 3: Figure S2.** Quantile–quantile (Q–Q) plots of the SNP distribution after quality control in GWAS for all tested meat quality traits. Datasets include 39,369 SNPs that were genotyped in the DLY population. In the Q–Q plots, -log_10_ (*P*) values of observed association statistics on the y-axis are compared to those expected under the hypothesis of no association on the x-axis. The solid line represents concordance between observed and expected values. The shaded region shows the 95% confidence interval based on the beta distribution. Genomic inflation factor, λ, is shown for each dataset.

**Additional file 4: Table** **S2.** Screening for mutations in the *PRKAG3* gene in six pigs with different genotypes (*AA*, *AG* and *GG*) at the GWAS tag SNP rs326377357. Fifty-three SNPs in *PRKAG3* were identified by comparative sequence analysis. Their positions on the Sscrofa11.1 pig genome assembly are shown. The grey lines highlight the 31 markers that were used to type the 610 DLY pigs.

**Additional file 5: Figure S3.** Haplotype diagram of 53 SNPs in six DLY individuals. By sequencing the *PRKAG3* gene in six DLY animals, 53 SNPs and nine haplotypes were identified, 31 of these SNPs indicated by solid triangles were selected and genotyped for the whole DLY population. The other 22 SNPs indicated by the empty triangles were not genotyped, but they were in complete linkage disequilibrium (LD) with selected markers of the same colour. The upper and lower triangles indicate the two alleles of a SNP. All alleles of haplotype 1 are represented by both the letters and upper triangles.

**Additional file 6: Table** **S3.** Information about the 73 SNPs selected and genotyped on the whole DLY population. Of the 73 genotyped markers, 31 marked with a grey line were derived from *PRKAG3* re-sequencing data, and the other 42 SNPs adjacent to *PRKAG3* were screened from online databases (e.g. the UCSC Genome Browser, NCBI and Ensembl), the literature and our own whole-genome sequence data of different European commercial pig breeds.

**Additional file 7: Figure S4.** Manhattans plots of GWAS for seven meat quality traits with increased marker density in the SSC15 QTL region. These traits include GP, pH, drip loss, Minolta L*, a*, b* and color score. The *PRKAG3* R200Q mutation was the most significant SNP across all seven meat traits.

**Additional file 8: Table** **S4.** Effects of four missense SNPs in *PRKAG3* on RG level in the replication cohort. The effects of *PRKAG3* G52S, I199V, L53P and R200Q mutations on RG level were assessed. G52S and I199V were not significantly associated with RG level (*P* = 0.97 and 0.053, respectively), whereas R200Q and L53P showed the same significant (*P* = 2.77 × 10^−9^) association with this trait.

**Additional file 9: Table** **S5.** Identification of parental origin (Landrace or Yorkshire) of the *RN*^−^ allele in DLY pigs. A haplotype-sharing analysis was conducted on 10 individuals in the 3.2-Mb region (117.6–120.8 Mb) upstream of the *RN*^−^ locus. The classical *RN*^−^ haplotypes are highlighted in blue. Obviously, the haplotypic fragments marked in yellow are shared between three DLY pigs and two Yorkshire pigs, while those marked in green are shared between DLY0800 and Landrace2612.

## Data Availability

The datasets used and analysed during the current study are available from the corresponding author on reasonable request.
