# Supplementary material for: Muscle glycogen level and occurrence of acid meat in commercial hybrid pigs are regulated by two low-frequency causal variants with large effects and multiple common variants with small effects
Source: Genet Sel Evol. 2019 Aug 23;51:46. doi: 10.1186/s12711-019-0488-0 (PMC6708195; doi:10.1186/s12711-019-0488-0)
Supplement: Supplementary file 1 — Additional file 1: Table S1. Primers used for sequencing the PRKAG3 gene. Fifteen pairs of primers were used to sequence the porcine PRKAG3 gene and identify variants. [file 12711_2019_488_MOESM1_ESM.docx]

**Table S1 Primers used for DNA sequencing of the *PRKAG3* gene**

| Primer | Sequence (5’ – 3’) |
| --- | --- |
| FP-1 | CCCCACCGATTCTCCTGTTT |
| RP-1 | GTTCGATCCCTGGCCTTGCC |
| FP-2 | TTTTTTCCTTTTTACGGTGG |
| RP-2 | TGGTGGAGTGGGATGGTTGG |
| FP-3 | TTCAAGCCCACTTCATACCT |
| RP-3 | ACAGAACCGCACAGACTCAC |
| FP-4 | CACCCAATCAGAGAGAACCC |
| RP-4 | AAGCAATTTGCCACAAATCA |
| FP-5 | CTTTGCCTTCTTTGTTCCGC |
| RP-5 | TCCTCCTGCCTTGTCCATCT |
| FP-6 | GGTTCCTAGTGGGATTCGTT |
| RP-6 | CCGCTCTGTTGGGGGGTTGT |
| FP-7 | TGGAGACAGCAGATGAGGAA |
| RP-7 | ACACAGCGAGAAGCACGGAA |
| FP-8 | GCACGCACCCCTTAATTGTC |
| RP-8 | CTGCTTCTTGCTGTCCCACA |
| FP-9 | TCACCCTCAACTGTTCTCTC |
| RP-9 | TCTTATGTTCTTCAATCTCG |
| FP-10 | CTTTGGGAGTTCCGTTGCTG |
| RP-10 | AGGGTGCCCTGGTTGGGTTG |
| FP-11 | CAGGGGGGCTGGGATGAAGG |
| RP-11 | AGGTGGATACGTGGGGTTCA |
| FP-12 | AAAAAGACTTAAAGGAACCA |
| RP-12 | CATCACACAGAGAGACACAC |
| FP-13 | GGAACACTTCATCTGCCTCT |
| RP-13 | TCCATCCATCTTCACACCAC |
| FP-14 | TCCCCCACAGGACAGGTAGT |
| RP-14 | CAAAGTGTTGTTAAGAAAAA |
| FP-15 | CTGTTGTTGTTTTTTGTTTA |
| RP-15 | ATGAGGTTTCAGGTTTGACC |
