# Supplementary figures and images for: Muscle glycogen level and occurrence of acid meat in commercial hybrid pigs are regulated by two low-frequency causal variants with large effects and multiple common variants with small effects
Source: Genet Sel Evol. 2019 Aug 23;51:46. doi: 10.1186/s12711-019-0488-0 (PMC6708195; doi:10.1186/s12711-019-0488-0)

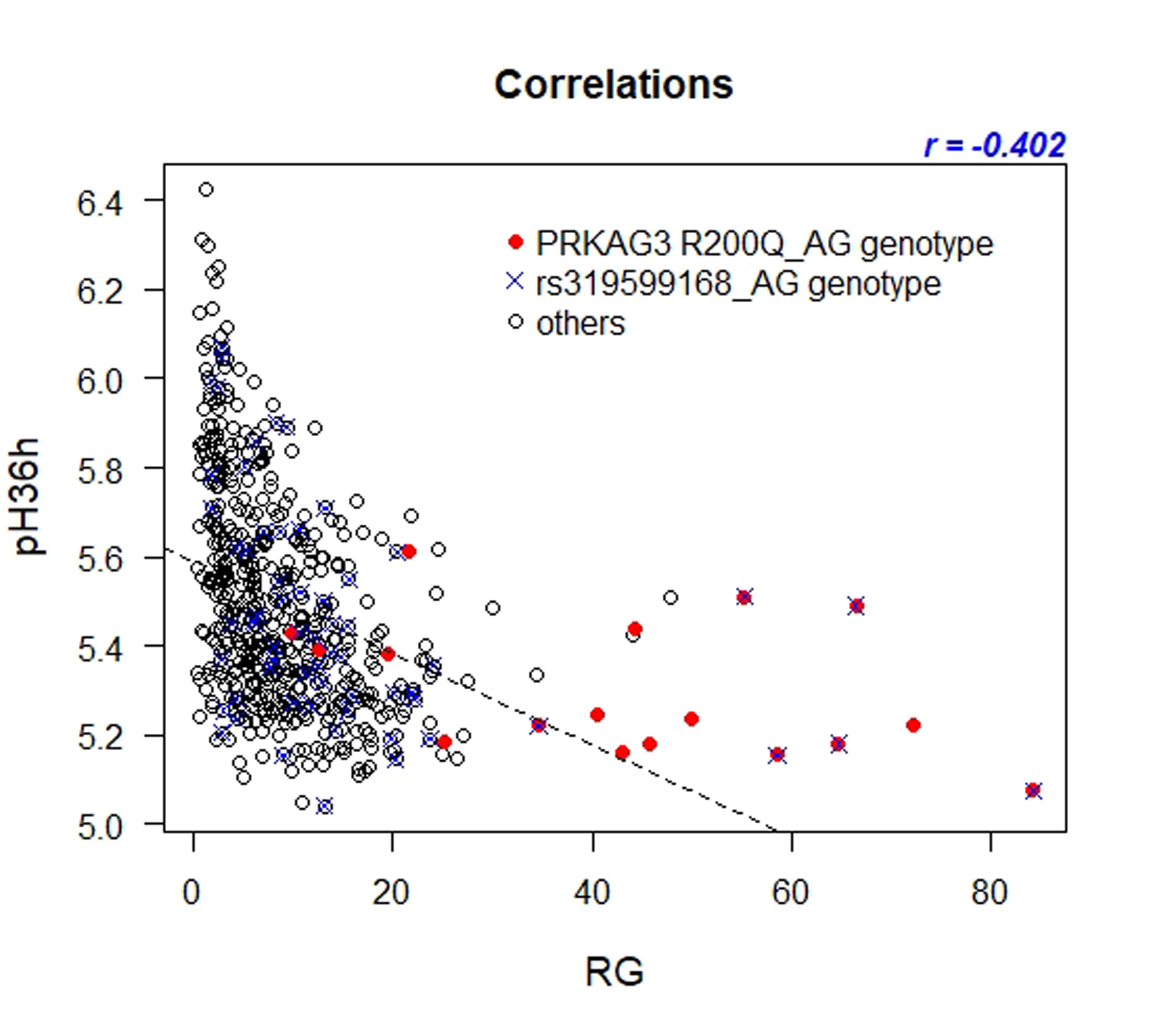

Supplement: Supplementary file 2 — Additional file 2: Figure S1. Correlation coefficients between RG level and pH 36 h. The x-axis and y-axis represent RG level and pH 36 h, respectively. Compared with most of the wild-type homozygotes, 17 individuals heterozygous at R200Q tended to have a higher RG level and lower pH 36 h, and six of them were also heterozygous at the GWAS tag SNP rs319599168. [file 12711_2019_488_MOESM2_ESM.tif]

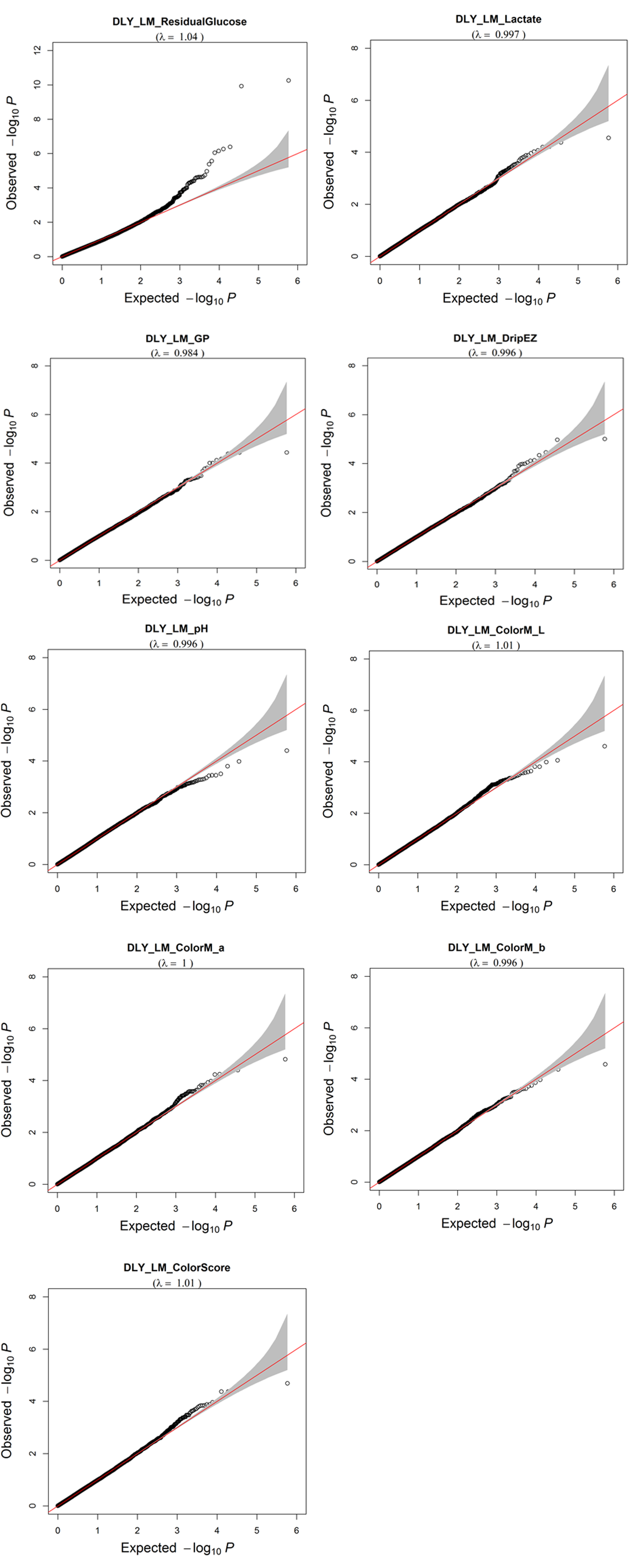

Supplement: Supplementary file 3 — Additional file 3: Figure S2. Quantile–quantile (Q–Q) plots of the SNP distribution after quality control in GWAS for all tested meat quality traits. Datasets include 39,369 SNPs that were genotyped in the DLY population. In the Q–Q plots, -log10 (P) values of observed association statistics on the y-axis are compared to those expected under the hypothesis of no association on the x-axis. The solid line represents concordance between observed and expected values. The shaded region shows the 95% confidence interval based on the beta distribution. Genomic inflation factor, λ, is shown for each dataset. [file 12711_2019_488_MOESM3_ESM.tif]

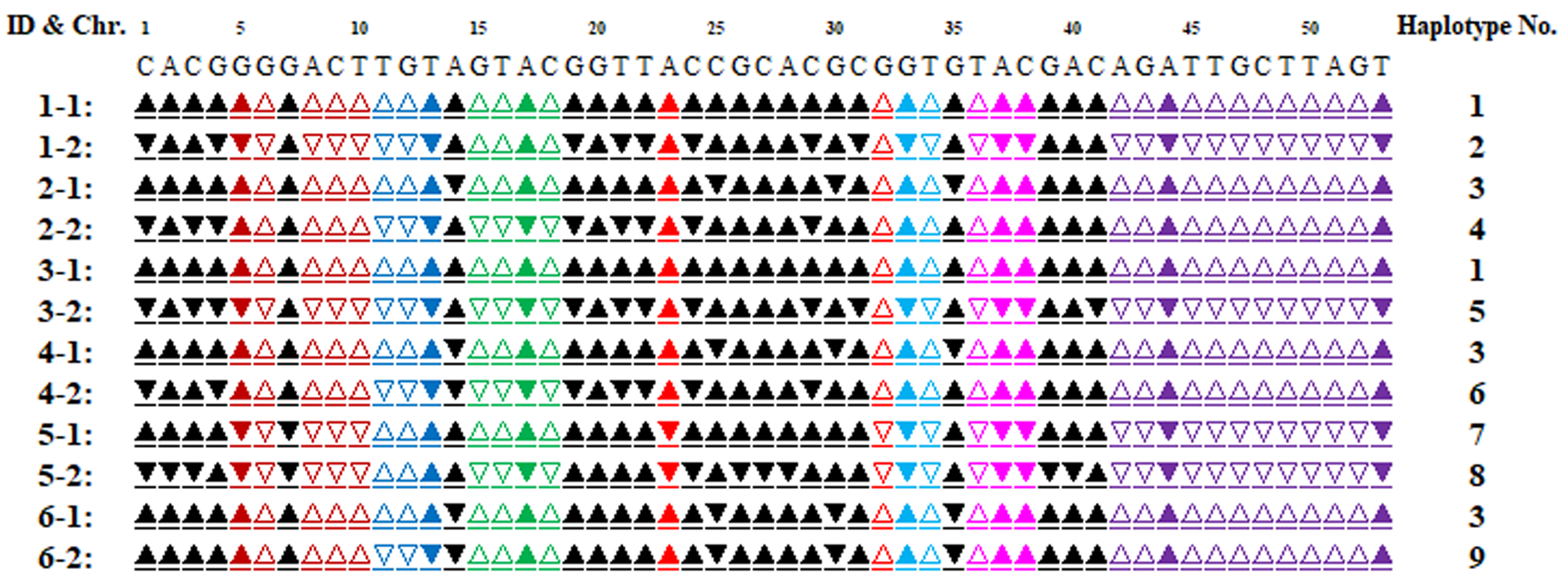

Supplement: Supplementary file 5 — Additional file 5: Figure S3. Haplotype diagram of 53 SNPs in six DLY individuals. By sequencing the PRKAG3 gene in six DLY animals, 53 SNPs and nine haplotypes were identified, 31 of these SNPs indicated by solid triangles were selected and genotyped for the whole DLY population. The other 22 SNPs indicated by the empty triangles were not genotyped, but they were in complete linkage disequilibrium (LD) with selected markers of the same colour. The upper and lower triangles indicate the two alleles of a SNP. All alleles of haplotype 1 are represented by both the letters and upper triangles. [file 12711_2019_488_MOESM5_ESM.tif]

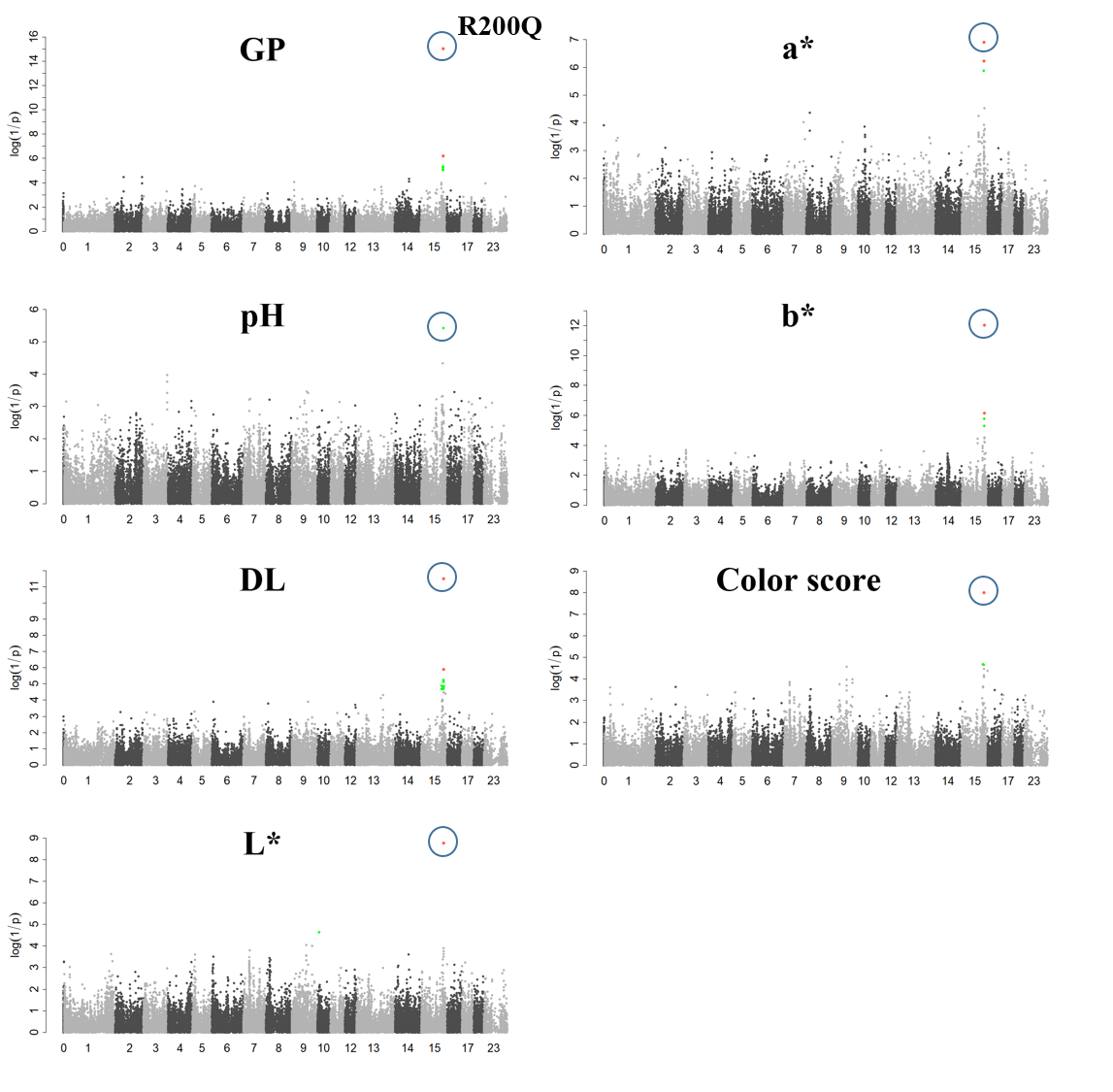

Supplement: Supplementary file 7 — Additional file 7: Figure S4. Manhattans plots of GWAS for seven meat quality traits with increased marker density in the SSC15 QTL region. These traits include GP, pH, drip loss, Minolta L*, a*, b* and color score. The PRKAG3 R200Q mutation was the most significant SNP across all seven meat traits. [file 12711_2019_488_MOESM7_ESM.tif]
