# Supplementary material for: Muscle glycogen level and occurrence of acid meat in commercial hybrid pigs are regulated by two low-frequency causal variants with large effects and multiple common variants with small effects
Source: Genet Sel Evol. 2019 Aug 23;51:46. doi: 10.1186/s12711-019-0488-0 (PMC6708195; doi:10.1186/s12711-019-0488-0)
Supplement: Supplementary file 4 — Additional file 4: Table S2. Screening for mutations in the PRKAG3 gene in six pigs with different genotypes (AA, AG and GG) at the GWAS tag SNP rs326377357. Fifty-three SNPs in PRKAG3 were identified by comparative sequence analysis. Their positions on the Sscrofa11.1 pig genome assembly are shown. The grey lines highlight the 31 markers that were used to type the 610 DLY pigs. [file 12711_2019_488_MOESM4_ESM.docx]

**Table S2 Screening for mutations in the *PRKAG3* gene in six pigs with different genotypes (*AA*, *AG* and *GG*) at the GWAS tag SNP rs326377357**

| **Num** | **Typed SNPs^1^** | **Position (bp)** | **No.1**  **(AA, 84.15)^2^** | **No.2**  **(AA, 64.63)** | **No.3**  **(AG, 49.93)** | **No.4**  **(AG, 45.81)** | **No.5**  **(GG, 7.31)** | **No.6**  **(GG, 6.11)** | **Consequence type** |
| --- | --- | --- | --- | --- | --- | --- | --- | --- | --- |
| 1 | *ts101*^▲^ | 120861378 | CT | CT | CT | CT | CT | CC | Intron |
| 2 | *ts100*^▲^ | 120861563 | AA | AA | AA | AA | AG | AA | Intron |
| 3 | *ts108*^▲^ | 120861788 | CC | CG | CG | CC | CG | CC | Intron |
| 4 | *ts107*^▲^ | 120861791 | GC | GC | GC | GC | GG | GG | Intron |
| 5 | *ts90*^▲^ | 120862382 | GA | GG | GA | GG | AA | GG | Intron |
| 6 |  | 120862470 | GA | GG | GA | GG | AA | GG | Intron |
| 7 | *ts97*^▲^ | 120862534 | GG | GG | GG | GG | AA | GG | Intron |
| 8 |  | 120862767 | AG | AA | AG | AA | GG | AA | Intron |
| 9 |  | 120862796 | CG | CC | CG | CC | GG | CC | Intron |
| 10 |  | 120862798 | TG | TT | TG | TT | GG | TT | Intron |
| 11 |  | 120863031 | TC | TC | TC | TC | TT | TC | Intron |
| 12 |  | 120863032 | GC | GC | GC | GC | GG | GC | Intron |
| 13 | *ts106*^▲^ | 120863040 | TC | TC | TC | TC | TT | TC | Intron |
| 14 | *ts105*^▲^ | 120863333 | AA | AG | AA | GG | AA | GG | Intron |
| 15 |  | 120863396 | GG | GC | GC | GC | GC | GG | Intron |
| 16 |  | 120863408 | TT | TC | TC | TC | TC | TT | Intron |
| 17 | *ts104*^▲^ | 120863444 | AA | AG | AG | AG | AG | AA | Intron |
| 18 |  | 120863462 | CC | CT | CT | CT | CT | CC | Intron |
| 19 | *R200Q*^▲^ | 120863533 | AG | AG | AG | AG | GG | GG | Exon/Missense_variant/R200Q |
| 20 | *I199V*^▲^ | 120863537 | GG | GG | GG | GG | GG | GG | Exon/Missense_variant/I199V |
| 21 | *194L*^▲^ | 120863552 | TC | TC | TC | TC | TT | TT | Exon/Synonymous_variant/194L |
| 22 | *193A*^▲^ | 120863553 | TC | TC | TC | TC | TT | TT | Exon/Synonymous_variant/193A |
| 23 | *ts96*^▲^ | 120863745 | AA | AA | AA | AA | GG | AA | Intron |
| 24 | *ts102*^▲^ | 120863777 | CT | CT | CT | CT | CT | CC | Intron |
| 25 | *ts92*^▲^ | 120863903 | CC | TC | CC | TC | CC | TT | Intron |
| 26 | *ts98*^▲^ | 120864329 | GG | GG | GG | GG | GA | GG | Intron |
| 27 | *ts99*^▲^ | 120864620 | CC | CC | CC | CC | CT | CC | Exon/Missense_variant/P134L |
| 28 | *ts48*^▲^ | 120864629 | AA | AA | AA | AA | AG | AA | Exon/Missense_variant/K131R |
| 29 | *ts42*^▲^ | 120864863 | CT | CT | CT | CT | CC | CC | Exon/Missense_variant/L53P |
| 30 | *G52S*^▲^ | 120865227 | GG | GA | GG | GA | GG | AA | Exon/Missense_variant/G52S |
| 31 | *T30N*^▲^ | 120865292 | AC | CC | AC | CC | CC | CC | Exon/Missense_variant/T30N |
| 32 |  | 120865479 | GG | GG | GG | GG | AA | GG | Intron |
| 33 | *ts89*^▲^ | 120865869 | GA | GG | GA | GG | AA | GG | Exon/Missense_variant/E47K |
| 34 |  | 120866041 | GT | TT | GT | TT | GG | TT | Intron |
| 35 | *ts91*^▲^ | 120866126 | GG | GA | GG | GA | GG | AA | Intron |
| 36 |  | 120866145 | TC | TT | TC | TT | CC | TT | Intron |
| 37 | *ts46*^▲^ | 120866366 | AG | AA | AG | AA | GG | AA | Intron |
| 38 | *ts45*^▲^ | 120866463 | CG | CC | CG | CC | GG | CC | Intron |
| 39 | *ts44*^▲^ | 120866528 | GG | GG | GG | GG | GA | GG | 5'UTR |
| 40 | *ts43*^▲^ | 120866618 | AA | AA | AA | AA | AG | AA | 5'UTR |
| 41 | *ts93*^▲^ | 120866787 | CC | CC | CT | CC | CC | CC | 5'UTR |
| 42 |  | 120866969 | GA | AA | GA | AA | GG | AA | Intron |
| 43 |  | 120867274 | AG | GG | AG | GG | AA | GG | Intron |
| 44 | *ts66*^▲^ | 120867300 | AG | AA | AG | AA | GG | AA | Intron |
| 45 |  | 120867323 | CT | TT | CT | TT | CC | TT | Intron |
| 46 |  | 120867326 | CT | TT | CT | TT | CC | TT | Intron |
| 47 |  | 120867374 | GT | GG | GT | GG | TT | GG | Intron |
| 48 |  | 120867433 | CT | CC | CT | CC | TT | CC | Intron |
| 49 |  | 120867441 | TG | TT | TG | TT | GG | TT | Intron |
| 50 |  | 120867471 | TG | TT | TG | TT | GG | TT | Intron |
| 51 |  | 120867511 | GA | AA | GA | AA | GG | AA | Intron |
| 52 |  | 120867573 | GA | GG | GA | GG | AA | GG | Intron |
| 53 | *ts88*^▲^ | 120867612 | CT | TT | CT | TT | CC | TT | Intron |

^1^Out of 53 SNPs identified in the *PRKAG3* gene, 31 SNPs with solid triangles here and in Figure S3 were used for genotyping the whole DLY pig population.

^2^the genotypes at the GWAS tag SNP rs326377357 and the residual glycogen (RG) values (μmol/g) of the selected animals.
