# Supplementary material for: Muscle glycogen level and occurrence of acid meat in commercial hybrid pigs are regulated by two low-frequency causal variants with large effects and multiple common variants with small effects
Source: Genet Sel Evol. 2019 Aug 23;51:46. doi: 10.1186/s12711-019-0488-0 (PMC6708195; doi:10.1186/s12711-019-0488-0)
Supplement: Supplementary file 6 — Additional file 6: Table S3. Information about the 73 SNPs selected and genotyped on the whole DLY population. Of the 73 genotyped markers, 31 marked with a grey line were derived from PRKAG3 re-sequencing data, and the other 42 SNPs adjacent to PRKAG3 were screened from online databases (e.g. the UCSC Genome Browser, NCBI and Ensembl), the literature and our own whole-genome sequence data of different European commercial pig breeds. [file 12711_2019_488_MOESM6_ESM.docx]

**Table S3 Information about the 73 SNPs selected and genotyped on the whole DLY population**

| **Num** | **Typed SNPs** | **Position on SSC15, bp** | **Alleles** | **Consequence** | **Amino_acids** | **Symbl** | **MAF** |
| --- | --- | --- | --- | --- | --- | --- | --- |
| 1 | *ts120* | 120487664 | G/T | intron_variant |  | *VIL1* | 0.365 |
| 2 | *ts121* | 120499557 | C/T | intron_variant |  | *USP37* | 0.140 |
| 3 | *ts122* | 120514099 | C/A | intron_variant |  | *USP37* | 0.250 |
| 4 | *ts123* | 120526170 | C/A | intron_variant |  | *USP37* | 0.324 |
| 5 | *ts124* | 120539095 | C/A | intron_variant |  | *USP37* | 0.463 |
| 6 | *ts125* | 120552752 | C/T | intron_variant |  | *USP37* | 0.463 |
| 7 | *ts126* | 120564003 | G/A | intron_variant |  | USP37 | 0.383 |
| 8 | *ts127* | 120576392 | T/C | intron_variant |  | *USP37* | 0.021 |
| 9 | *ts128* | 120589211 | G/T | intron_variant |  | *USP37* | 0.500 |
| 10 | *ts129* | 120601305 | T/C | intron_variant |  | *CNOT9* | 0.248 |
| 11 | *ts139* | 120605203 | T/A | intron_variant |  | *CNOT9* | 0.137 |
| 12 | *ts130* | 120613338 | C/T | intron_variant |  | *CNOT9* | 0.001 |
| 13 | *ts138* | 120619039 | T/C | intron_variant |  | *CNOT9* | 0.312 |
| 14 | *ts49* | 120659632 | C/T | intron_variant |  | *PLCD4* | 0.310 |
| 15 | *ts136* | 120663966 | C/T | intron_variant |  | *PLCD4* | 0.173 |
| 16 | *ts137* | 120669086 | T/C | intron_variant |  | *PLCD4* | 0.423 |
| 17 | *ts85* | 120699048 | G/T | intron_variant |  | *ZNF142* | 0.082 |
| 18 | *ts111* | 120704411 | T/C | intron_variant |  | *ZNF142* | 0.376 |
| 19 | *ts114* | 120722385 | T/C | intron_variant |  | *STK36* | 0.208 |
| 20 | *ts113* | 120727814 | T/C | intron_variant |  | *STK36* | 0.043 |
| 21 | *ts86* | 120745901 | A/C | missense_variant | H1222Q | *STK36* | 0.369 |
| 22 | *ts131* | 120754929 | T/G | intergenic_variant |  | *-* | 0.010 |
| 23 | *ts132* | 120755917 | C/T | intron_variant |  | *CFAP65* | 0.070 |
| 24 | *ts109* | 120773427 | T/C | synonymous_variant | 81F | *TTLL4* | 0.094 |
| 25 | *ts116* | 120779196 | T/C | intron_variant |  | *TTLL4* | 0.208 |
| 26 | *ts115* | 120781563 | G/A | intron_variant |  | *TTLL4* | 0.095 |
| 27 | *ts110* | 120785926 | C/T | intron_variant |  | *TTLL4* | 0.094 |
| 28 | *ts50* | 120801239 | T/C | intergenic_variant |  | *-* | 0.000 |
| 29 | *ts133* | 120820631 | G/A | intron_variant |  | *CPY27A1* | 0.374 |
| 30 | *ts87* | 120831041 | A/G | intron_variant |  | *CPY27A1* | 0.300 |
| 31 | *ts84* | 120839332 | T/C | intron_variant |  | *CYP27A1* | 0.148 |
| 32 | *ts117* | 120839939 | A/G | intron_variant |  | *CYP27A1* | 0.463 |
| 33 | *ts112* | 120850466 | T/C | intron_variant |  | *PRKAG3* | 0.000 |
| 34 | *ts101* | 120861378 | C/T | intron_variant |  | *PRKAG3* | 0.095 |
| 35 | *ts100* | 120861563 | A/G | intron_variant |  | *PRKAG3* | 0.094 |
| 36 | *ts108* | 120861788 | C/G | intron_variant |  | *PRKAG3* | 0.161 |
| 37 | *ts107* | 120861791 | G/C | intron_variant |  | *PRKAG3* | 0.042 |
| 38 | *ts90* | 120862382 | G/A | intron_variant |  | *PRKAG3* | 0.371 |
| 39 | *ts97* | 120862534 | A/G | intron_variant |  | *PRKAG3* | 0.370 |
| 40 | *ts106* | 120863040 | T/C | intron_variant |  | *PRKAG3* | 0.097 |
| 41 | *ts105* | 120863333 | G/A | intron_variant |  | *PRKAG3* | 0.383 |
| 42 | *ts104* | 120863444 | A/G | intron_variant |  | *PRKAG3* | 0.109 |
| 43 | *R200Q* | 120863533 | G/A | missense_variant | R200Q | *PRKAG3* | 0.014 |
| 44 | *I199V* | 120863537 | G/A | missense_variant | I199V | *PRKAG3* | 0.220 |
| 45 | *194L* | 120863552 | T/C | synonymous_variant | 194L | *PRKAG3* | 0.109 |
| 46 | *193A* | 120863553 | T/C | synonymous_variant | 193A | *PRKAG3* | 0.109 |
| 47 | *ts96* | 120863745 | G/A | intron_variant |  | *PRKAG3* | 0.102 |
| 48 | *ts102* | 120863777 | C/T | intron_variant |  | *PRKAG3* | 0.109 |
| 49 | *ts92* | 120863903 | T/C | intron_variant |  | *PRKAG3* | 0.196 |
| 50 | *ts98* | 120864329 | G/A | intron_variant |  | *PRKAG3* | 0.274 |
| 51 | *ts99* | 120864620 | C/T | missense_variant | P134L | *PRKAG3* | 0.274 |
| 52 | *ts48* | 120864629 | A/G | missense_variant | K131R | *PRKAG3* | 0.093 |
| 53 | *ts42* | 120864863 | C/T | missense_variant | L53P | *PRKAG3* | 0.014 |
| 54 | *ts95* | 120865011 | C/T | intron_variant |  | *PRKAG3* | 0.094 |
| 55 | *ts47* | 120865260 | G/A | missense_variant | V41I | *PRKAG3* | 0.093 |
| 56 | *G52S* | 120865227 | G/A | missense_variant | G52S | *PRKAG3* | 0.400 |
| 57 | *T30N* | 120865292 | C/A | missense_variant | T30N | *PRKAG3* | 0.325 |
| 58 | *ts89* | 120865869 | G/A | missense_variant | E47K | *PRKAG3* | 0.371 |
| 59 | *ts91* | 120866126 | A/G | intron_variant |  | *PRKAG3* | 0.404 |
| 60 | *ts46* | 120866366 | A/G | 5_prime_UTR_variant |  | *PRKAG3* | 0.369 |
| 61 | *ts45* | 120866463 | C/G | 5_prime_UTR_variant |  | *PRKAG3* | 0.369 |
| 62 | *ts44* | 120866528 | G/A | 5_prime_UTR_variant |  | *PRKAG3* | 0.093 |
| 63 | *ts43* | 120866618 | A/G | 5_prime_UTR_variant |  | *PRKAG3* | 0.093 |
| 64 | *ts93* | 120866787 | C/T | 5_prime_UTR_variant |  | *PRKAG3* | 0.070 |
| 65 | *ts68* | 120866889 | C/T | 5_prime_UTR_variant |  | *PRKAG3* | 0.369 |
| 66 | *ts67* | 120866977 | A/G | upstream_gene_variant |  | *PRKAG3* | 0.000 |
| 67 | *ts66* | 120867300 | A/G | upstream_gene_variant |  | *PRKAG3* | 0.136 |
| 68 | *ts88* | 120867612 | T/C | upstream_gene_variant |  | *PRKAG3* | 0.136 |
| 69 | *ts51* | 120869774 | A/G | intron_variant |  | *CFAP65* | 0.369 |
| 70 | *ts135* | 120921966 | C/T | intron_variant |  | *CFAP65* | 0.048 |
| 71 | *ts134* | 120959046 | C/T | intron_variant |  | *CFAP65* | 0.100 |
| 72 | *ts119* | 120988251 | A/G | intron_variant |  | *CFAP65* | 0.298 |
| 73 | *ts118* | 120993428 | C/G | intron_variant |  | *CFAP65* | 0.002 |
