# Supplementary material for: Muscle glycogen level and occurrence of acid meat in commercial hybrid pigs are regulated by two low-frequency causal variants with large effects and multiple common variants with small effects
Source: Genet Sel Evol. 2019 Aug 23;51:46. doi: 10.1186/s12711-019-0488-0 (PMC6708195; doi:10.1186/s12711-019-0488-0)
Supplement: Supplementary file 8 — Additional file 8: Table S4. Effects of four missense SNPs in PRKAG3 on RG level in the replication cohort. The effects of PRKAG3 G52S, I199V, L53P and R200Q mutations on RG level were assessed. G52S and I199V were not significantly associated with RG level (P = 0.97 and 0.053, respectively), whereas R200Q and L53P showed the same significant (P = 2.77 × 10^−9^) association with this trait. [file 12711_2019_488_MOESM8_ESM.docx]

**Table S4 Effects of four missense SNPs in *PRKAG3* on RG level in the replication cohort**

| **SNPs** | **Phenotype^1^ (N)** | | | **MAF^2^** | ***P*-value** |
| --- | --- | --- | --- | --- | --- |
|  | **AA(N)** | **AB (N)** | **BB (N)** |  |  |
| G52S | 1.03 ± 1.05 (71) | 0.92 ± 0.86 (56) | 1.15 ± 0.77 (22) | 0.34 | 0.97 |
| I199V | 0.52 ± 0.49 (10) | 1.08 ± 0.93 (70) | 1.02 ± 0.98 (69) | 0.30 | 0.053 |
| L53P | na. | 4.04 ± 1.22 (3) | 0.95 ± 0.82 (146) | 0.01 | 2.77×10^-9^ |
| R200Q | na. | 4.04 ± 1.22 (3) | 0.95 ± 0.82 (146) | 0.01 | 2.77×10^-9^ |

^1^The phenotypic values of different genotypes (A and B represent a pair of allele at a locus) are shown as mean ± standard deviation. na, not available.

^2^minor allele frequency.
